# *Ycasd* – a tool for capturing and scaling data from graphical representations

**DOI:** 10.1186/1471-2105-15-219

**Published:** 2014-06-25

**Authors:** Arnd Gross, Sibylle Schirm, Markus Scholz

**Affiliations:** 1 Institute for Medical Informatics, Statistics and Epidemiology, University of Leipzig, Haertelstrasse 16-18, 04107 Leipzig, Germany; 2 LIFE – Leipzig Research Center for Civilization Diseases, University of Leipzig, Philipp-Rosenthal-Strasse 27, 04103 Leipzig, Germany

## Abstract

**Background:**

Mathematical modelling of biological processes often requires a large variety of different data sets for parameter estimation and validation. It is common practice that clinical data are not available in raw formats but are provided as graphical representations. Hence, in order to include these data into environments used for model simulations and statistical analyses, it is necessary to extract them from their presentations in the literature. For this purpose, we developed the freely available open source tool *ycasd*. After establishing a coordinate system by simple axes definitions, it supports convenient retrieval of data points from arbitrary figures.

**Results:**

After describing the general functionality and providing an overview of the programme interface, we demonstrate on an example how to use *ycasd*. A major advantage of *ycasd* is that it does not require a certain input file format to open and process figures. All options of *ycasd* are accessible through a single window which eases handling and speeds up data extraction. For subsequent processing of extracted data points, results can be formatted as a *Matlab* or an *R* matrix. We extensively compare the functionality and other features of *ycasd* with other publically available tools. Finally, we provide a short summary of our experiences with *ycasd* in the context of modelling.

**Conclusions:**

We conclude that our tool is suitable for convenient and accurate data retrievals from graphical representations such as papers. Comparison of tools reveals that *ycasd* is a good compromise between easy and quick capturing of scientific data from publications and complexity. Our tool is routinely applied in the context of biological modelling, where numerous time series data are required to develop models. The software can also be useful for other kinds of analyses for which published data are required but are not available in raw formats such as systematic reviews and meta-analyses.

## Background

In medical literature, data of other groups are often not available in their raw formats but are presented in figures such as scatter plots, box plots, time series data or derived statistics such as Kaplan-Meier curves. However, incorporating these data in research projects is often necessary to compare one’s own results with those of the literature. This especially applies for systems-biological modelling for which time series data under different clinical conditions are required to calibrate the models and to validate their predictions. In order to include these data into environments used for model simulations, it is necessary to extract them from the presentations chosen in the literature. To support this task, we developed the tool *ycasd* (***y*** *casd***c**aptures **a**nd **s**cales **d**ata) which can capture data from many kinds of graphical representations. The problem of skewed diagrams obtained from scanning papers is also addressed. For a typical example see Additional file 1.

The development of *ycasd* was motivated by our own work in the field of modelling blood formation. Time series data of numerous blood parameters are required for parameterisation and validation of our models of human thrombopoiesis [1], erythropoiesis [2], granulopoiesis under chemotherapy and growth-factor applications [3] and for other models currently under development.

Since we believe that our tool is helpful especially for other research groups interested in dynamical modelling or meta-analyses, we aim at publishing it as an open source and freely available software. In the present paper, we describe the functionality and the interface of the programme, compare it with those of other publically available tools and provide a quick introduction to its use on the basis of example figures.

## Implementation

In this section, we describe the mathematics behind *ycasd* and corresponding practical limitations.

### Vector calculations

**Figure 1:**
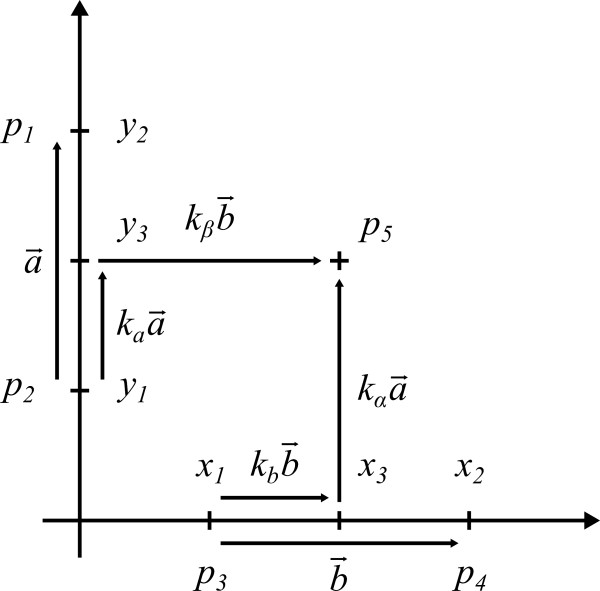
**Vector representation.** Pixels *p*_1_ to *p*_4_ define the coordinate system and correspond to axis intercepts *y*_2_, *y*_1, _*x*_1_, and *x*_2_, respectively. For each new pixel *p*_5_, coordinates *x*_3, _y_3_ will be calculated by *ycasd*.

Two pairs of pixels define the vectors of the axes provided that these vectors are linearly independent. Setting values for the coordinates of the pixels and specifying the scaling of the axes (linear or logarithmic) define the coordinate system. We aim to calculate the coordinates for any arbitrary fifth pixel regarding to this coordinate system. We restrict to a linear scaling of the axes in the following since logarithmic scaling can be traced back to this case by a simple transformation. As depicted in Figure 1, pixels *p*_1_,*p*_2_ and *p*_3_,*p*_4_ correspond to axis intercepts *y*_2_,*y*_1_ and *x*_1_,*x*_2_, respectively. Vectors $\vec{a}$ and $\vec{b}$ are coordinate vectors of the y- and x-axis respectively. When considering pixels, we can set

(1) $${\vec{p}}_{i}=\left(\begin{array}{c} p_{i1}\\ p_{i2} \end{array}\right),\;p_{i1},p_{i2}\in N\;\mathrm{for}\;i=1,2,\ldots ,5$$

(2) $$\vec{a}={\vec{p}}_{1}-{\vec{p}}_{2}=\left(\begin{array}{c} a_{1}\\ a_{2} \end{array}\right),\;a_{1},a_{2}\in Z$$

(3) $$\vec{b}={\vec{p}}_{4}-{\vec{p}}_{3}=\left(\begin{array}{c} b_{1}\\ b_{2} \end{array}\right),\;b_{1},b_{2}\in Z$$

For each new captured pixel *p*_5_, the corresponding coordinate pair *x*_3_,*y*_3_ is calculated by estimating the intercept on each axis defined by a line, which is parallel to the respective other axis crossing *p*_5_. We assume that vectors $k_{\alpha }\vec{a}$ and $k_{\beta }\vec{b}$ are pointing in the same direction as the axes to account for rotated or linearly skewed figures, e.g. as result of scanning. Orthogonality of both axes is not required. The system of resulting equations is as follows

(4) $${\vec{p}}_{2}+k_{a}\vec{a}={\vec{p}}_{5}-k_{\beta }\vec{b}$$

(5) $${\vec{p}}_{3}+k_{b}\vec{b}={\vec{p}}_{5}-k_{\alpha }\vec{a}$$

We aim to determine the coefficients *k*_*a*_ and *k*_*b*_. Solving the equations yields

(6) $$k_{a}=\frac{\left(p_{51}-p_{21}\right)b_{2}-\left(p_{52}-p_{22}\right)b_{1}}{a_{1}b_{2}-a_{2}b_{1}}$$

(7) $$k_{b}=\frac{\left(p_{51}-p_{31}\right)a_{2}-\left(p_{52}-p_{32}\right)a_{1}}{a_{2}b_{1}-a_{1}b_{2}}$$

A solution exists if *a*_1_*b*_2_ ≠ *a*_2_*b*_1_, i.e. if both axes are linearly independent. The coordinates *x*_3_,*y*_3_ can now be calculated:

(8) $$x_{3}=x_{1}+k_{b}\left(x_{2}-x_{1}\right)$$

(9) $$y_{3}=y_{1}+k_{a}\left(y_{2}-y_{1}\right)$$

If the y axis is logarithmically scaled, *y*_3_ can be determined by

(10) $$y_{3}^{\text{log}}=y_{1}{\left(\frac{y_{2}}{y_{1}}\right)}^{k_{a}}\;\mathrm{for}\;y_{1},y_{2}>0$$

The clicking error is defined as the deviation of the estimated coordinates if a neighbouring pixel is selected. It can be estimated in units of each axis by the ratios of the axis interval lengths and pixel distances, i.e.

(11) $$x_{\mathrm{err}}=\frac{\left|x_{1}-x_{2}\right|}{\sqrt{b_{1}^{2}+b_{2}^{2}}}$$

(12) $$y_{\mathrm{err}}=\frac{\left|y_{1}-y_{2}\right|}{\sqrt{a_{1}^{2}+a_{2}^{2}}}$$

*y*_err_ is only valid for non-logarithmically scaled y axes.

### Limitations

The assumption that $k_{\alpha }\vec{a}$ and $k_{\beta }\vec{b}$ are always parallel to the y and x axis is simple. Although more elaborated methods for calculation of coordinate pairs *x*_3_, *y*_3_ are conceivable to account for non-linearly distorted figures (by scanning), our approach allows for rotation and linear skewness and is straightforward to implement into a *C* programme. If the assumption of linear skewness is inadequate, raw pixel data displayed by the software in case of incomplete axes definition can be used to perform own calculations of coordinates.

Despite of the fact that *ycasd* is developed for Microsoft Windows, it can be used under Linux with *Wine*[4]. Mouse events are tracked by Windows API (Application Programming Interface) calls, therefore, only the graphical output of other Windows programmes running in *Wine* can be captured. As an example, one may use *Ghostscript* and *GSview* for Windows [5] for displaying figures in PDFs under Linux.

More technical limitations of the programme can be found in a readme file which is part of the *ycasd* package provided as Additional file 2.

## Results

### General functionality

Our freely available software tool *ycasd* supports capturing data points from figures of electronically available or scanned publications in order to make these data accessible by other software such as environments for data analysis or model simulations. Data capturing is possible for all kind of figures, which can be displayed on the screen under Microsoft Windows. It only requires the definition of two points for each of the axes by clicking and setting their corresponding coordinates. For each subsequent click on the figure, a coordinate pair is estimated by vector calculations considering axes definition. Vector calculations allow for capturing data points from rotated or skewed figures as a result of scanning papers. One can display the calculated coordinates either as a *Matlab* or an *R* matrix with adjustable accuracy.

For quick use, a guide is shown in the output box after starting the programme. This quick guide is read from a text file, which is part of the *ycasd* package, see Additional file 2. During runtime, the status bar shows useful information, too. Hotkeys for major functions are available.

### Overview of the programme interface

The interface of *ycasd* consists of a single window. It is divided in areas corresponding to different actions which are briefly described below. For each action we present a description, corresponding control options and their usage on a working example.

### Define axes

#### Description

**Figure 2:**
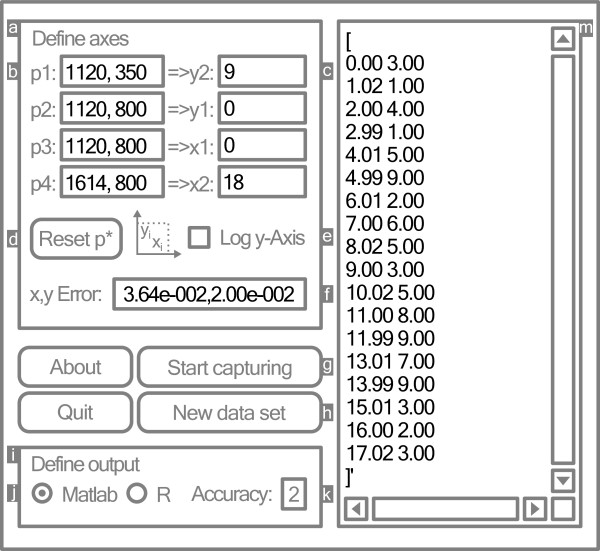
***Ycasd *interface window.** Representation of *ycasd* interface window. Settings apart from default as well as outputs are shown in black. White letters on grey background are labels for: “Define axes” area **(a)**, Pixels p1 to p4 corresponding to axis intercepts **(b)**, Values corresponding to axis intercepts **(c)**, “Reset p*” button **(d)**, Logarithmic y-Axis selector **(e)**, Estimated clicking error **(f)**, “Start/Stop capturing” button **(g)**, “New data set” button **(h)**, “Define output” area **(i)**, Output matrix type selector **(j)**, Output accuracy **(k)**, Output text box **(m)**.

All settings regarding axes definition are located in the “Define axes” area (Label “a” in Figure 2).

#### List of controls

• **Axis intercepts p1-p4** (Label “b” in Figure 2) are defined by four pixels on the screen. Pixel pair p1, p2 and p3, p4 belong to the y-axis and x-axis, respectively. Points should be chosen in such a way that they span a large distance on each axis which improves accuracy of derived coordinates.

• **Coordinates y2, y1, x1, x2** of axes (Label “c” in Figure 2) must be defined in the next step and correspond to the intercepts p1 to p4 selected in the first step. The order y2, y1, x1, x2 was chosen to minimise mouse distances when clicking on axes beginning on the upper left and moving to the lower right.

• Reset p* button (Label “d” in Figure 2) clears the four pixels defining the axis intercepts. Subsequently captured pixels redefine the axis intercepts.

• **Log y-Axis** selector (Label “e” in Figure 2) supports logarithmic scaling of the y-Axis.

• **x, y Error** (Label “f” in Figure 2) indicates the clicking error in units of the corresponding axis.

#### Working example

**Figure 3:**
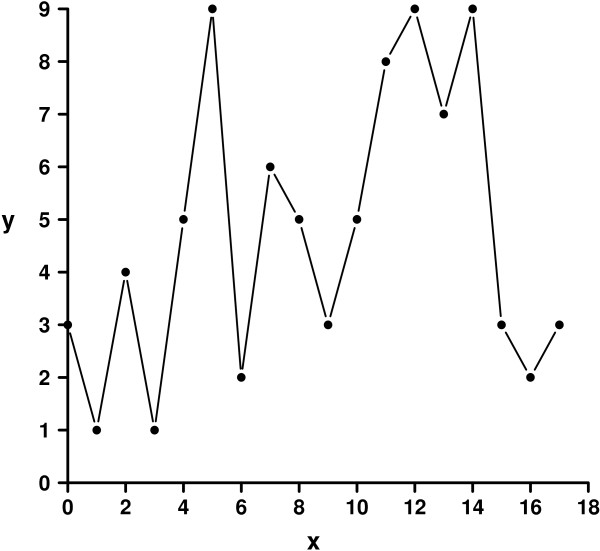
Sample figure.

Step 1. Display Figure 3 on the screen or open “example.pdf” (see also Additional file 2) with your preferred PDF reader, e.g. *GSview*[5].

Step 2. Define y2, y1, x1 and x2 by typing in the values 9, 0, 0 and 18, respectively.

### Capture data

#### Description

Pixels are captured from the (raw) screen independently of viewer or file format. If the four pixels of the axis intercepts are unset, i.e. at programme start or after “Reset p*”, they will be captured first. The coordinates of subsequently captured pixels will be scaled regarding axes definition and displayed in the output box. The quick guide shown after starting the software automatically disappears when results from capturing are available.

#### List of controls

• **Start capturing** button (Label “g” in Figure 2) activates data capturing. While capturing, the “Start capturing” button is re-labelled as “Stop capturing” allowing to stop the data retrieval.

• **New data set** button (Label “h” in Figure 2) clears the output.

#### Working example

Step 3. Push “Start capturing” and capture the four pixels p1 to p4 defining the axis intercepts by simply left clicking p1 to p4 as depicted in Figure 1, i.e. intercepts at 9 and 0 on the y-axis and intercepts at 0 and 18 on the x-axis.

Step 4. While clicking on every data point of Figure 3, the coordinates are calculated with respect to the axes definition and displayed in the output box. The results should be similar to the values presented in Figure 2.

### Define output

#### Description

Settings in the “Define output” area (Label “i” in Figure 2) control how results are displayed. Each time output style or axes definition is changed, output coordinate pairs will be re-calculated accordingly. If the axes definition is incomplete, raw pixel data will be presented in the output.

#### List of controls

• **Output matrix** selector (Label “j” in Figure 2) formats the output as n-by-two matrix either for the numerical software *Matlab*[6] or the statistical software package *R*[7].

• **Output Accuracy** (Label “k” in Figure 2) defines the number of digits after the decimal point of the output.

• **Output** box (Label “m” in Figure 2) contains the output. Resulting coordinate pairs can be edited as follows: Placing the cursor after an output coordinate allows insertion of pairs. If the cursor is placed within a coordinate pair, the whole pair will be selected. It can be overwritten by a new pair or deleted by pressing the “delete” or “backspace” key. If the selection of a pair is ambiguous or incomplete, deletion as well as capturing of new data points is blocked to prevent undesired results.

#### Working example

Step 5. Try to modify the output style, e.g. by changing it to an *R* matrix and increasing the output accuracy to 3.

Step 6. Overwrite the first output coordinate pair by placing the cursor within the first pair and re-capture data point (0,3).

Step 7. Push “Stop capturing” to inactivate capturing of data. The label of the button is replaced by “Start capturing” again allowing to continue data retrieval.

### Tool survey

#### Tool selection

Since many software tools are available for data point extraction from figures, we conducted a comprehensive tool survey to compare these tools with *ycasd*. An introduction and a list of tools for analysing scanned graphs is given in Wikipedia [8]. We considered only software for which source code is available and which fulfil the requirements of Free Software [9]. Six tools were tested and compared with *ycasd*: *Dexter*[10], *digitize*[11], *Engauge Digitizer*[12], *g3data*[13], *Plot Digitizer*[14] and *WebPlotDigitizer*[15]. All programmes are protected by the GNU General Public License (GPL) version 2 (GPLv2) or version 3 (GPLv3) or by the Lesser GNU General Public License (LGPL) version 2 (LGPLv2). For details of the GPL, see [16]. These tools differ in handling and functionality. Therefore we consider different categories for comparison purposes as described below (see also Table 1).

**Table 1:** Tool survey

	**Dexter**	**Digitize**	**Engauge Digitizer**	**g3data**	**Plot Digitizer**	**WebPlotDigitizer**	**ycasd**
License	GPLv2	GPLv2	GPLv2	GPLv2	GPLv2, LGPLv2	GPLv3	GPLv3
Platform	Java	R	Linux, Windows	Linux, Windows	Java	HTML5	Windows
Version	0.5a	0.0.2	5.1	1.5.4	2.6.4	2.6	1.5
Documentation	Sufficient	Sparse	Extensive	Sparse	Sufficient	Extensive	Sufficient
Single window	Yes	No	No	Yes	No	No	Yes
Figure import	Graphic	Graphic	Graphic, clipboard	Graphic	Graphic	Graphic, clipboard (Chrome)	not necessary
Diagram type	Cartesian	Cartesian	Raw pixel, Cartesian, polar, map	Cartesian	Raw pixel, Cartesian	Raw pixel, Cartesian, polar, ternary, map	Raw pixel, Cartesian
Calibration points	4	4	3	4	4 (optional 3)	4	4
Log Axis	Both	n.a.	Both	Both	Both	Both	y-axis
Magnifier	Yes	No	No	Yes	No	Yes	No
Data point correction	Delete	Not available	Insert, delete, shift	Delete last point	Insert, delete, shift (only XML)	Delete	Insert, delete, overwrite
Axis correction	Shift	Re-capture	Shift	Re-capture	n.a.	Re-capture	Re-capture
Distortion correction level	Rotated, skewed	None	Rotated, skewed	Rotated, skewed	Rotated	Rotated, skewed	Rotated, skewed
Output format	Delimiter	R	Delimiter	Delimiter	Delimiter, xml	Delimiter, plotly	Matlab, R

Comparison of tools regarding different categories. Not supported functions are denoted as “n.a.” (not available).

#### Platform and version

Platform independence is an advantage when providing software. *Dexter* and *Plot Digitizer* are implemented in Java which is available for all common operating systems such as Linux, Mac OS X or Windows. Similarly *R,* and with it, the *R* package *digitize* is available for different operating systems. *WebPlotDigitizer* is based on HTML5 which is supported by all popular browsers. Hence, these tools can be considered as independent of the operating system.

In contrast, *Engauge Digitizer*, *g3data* and *ycasd* must be compiled for different operating systems. In principle, when the source code is provided, this is possible if an appropriate compiler is available, e.g. the GNU Compiler Collection [17] for Linux, MAC OS X and Windows. But compiling fails if certain system dependent libraries are required, such as mouse tracking used by *ycasd*. For windows programmes, this problem can be handled by *Wine*[4]. On the other hand, it is challenging to build a programme from the most recent version of source code especially if non-default libraries are referenced or a certain library version is required as we experienced for *Engauge Digitize*r and *g3data*. Therefore, we decided to use the most recently available binaries for both tools. The versions of all tested programmes are shown in Table 1.

#### Documentation and handling

In preparation for data capturing from figures we studied the documentations of the tools which are either attached to the programme packages or accessible online. *Digitize* provides only a description of function parameters via the R help system. For *g3data,* we found only a description of command line parameters in the Linux man pages. We evaluate these documentations as “sparse”. *Dexter*, *Plot Digitizer* and *ycasd* attached at least one document explaining how to calibrate axes, extract data points and access primary functions (documentation denoted as “sufficient”). Additionally, *Engauge Digitizer* and *WebPlotDigitizer* provide multiple sources for help as FAQs, tutorials or videos (denoted as “extensive”).

Handling of tools refers to accessibility of major functions. For *ycasd* all functions are accessible via a single window which speeds up the data retrieval. In this sense, only *Dexter* and *g3data* are similarly effective. The other tools are more complex containing sub-menus or sub–windows.

#### Figure import and types of supported diagrams

In contrast to *ycasd*, all other tools directly import the graphic file to be analysed. As different graphic file formats were supported, we converted Figure 3 to a JPG (Joint photographic experts group file, see Additional file 3), which could be processed by all programmes and serves for testing. Additionally, *Engauge Digitizer* supports importing images from clipboard which facilitates analysis of screenshots. *WebPlotDigitizer* supports importing images from clipboard only when *Google Chrome* is used. A major advantage of *ycasd* is that it does not depend on a certain file format.

In addition to analysing simple Cartesian coordinates, *Engauge Digitizer*, *Plot Digitizer*, *WebPlotDigitizer* and *ycasd* are able to export raw pixel data which is useful for own calculations of coordinates. Polar plots and (geographical) maps can be analysed by *Engauge Digitizer* and *WebPlotDigitizer*. The latter even supports data extraction from ternary plots.

#### Axes calibration

Almost all tools necessitate specification of four points for axes calibration, i.e. two points per axis. In this case, only the corresponding intercepts are required. *Engauge Digitizer* is based on another concept. Here, three points with full coordinates must be specified. Using *Plot Digitizer*, one can choose whether to share one axis point or not. For *digitize*, *g3data*, *WebPlotDigitizer* and *ycasd*, this can be imitated by selecting the second point used for axes calibration twice.

Sometimes one or both axes of Cartesian plots are logarithmically scaled. Since *digitize* retrieves coordinates from a plot by a simple *R* function, it does not consider any logarithmic transformations. *Ycasd* supports only logarithmic scaling of the y-axis, because the x-axis refers to time in our main field of application. The other tools facilitate logarithmic scaling of both axes.

#### Retrieval of data points

Retrieval of data points by clicking each point is the most cumbersome part of the work. Aiming at the centre of a data point is easier when a magnifier is provided as implemented by *Dexter*, *g3data* and *WebPlotDigitizer*. If no magnifier is available, the figure can be up-scaled either by using an option (*Engauge Digitizer*, *Plot Digitizer*) or by manually adjusting the figure window (*digitize*, *ycasd*).

Another important issue are corrections necessary during the data retrieval process. For data points, *Engauge Digitizer* and *Plot Digitizer* present marks which can be shifted, deleted or inserted. However, *Plot Digitizer* only allows corrections as long as results are displayed as XML outputs. *Dexter* or *WebPlotDigitizer* feature deletion of single data points. Only *ycasd* supports deletion, insertion and overwriting of coordinate pairs by re-capturing selected data points. For correcting an arbitrary data point under *g3data* or *digitize* it is often more convenient to re-capture all points because *g3data* supports only removal of the last captured point. Correcting outputs of *digitize* implies that the data points must be manipulated within R.

Correcting axes without re-capturing all data points is also desirable. *Dexter* and *Engauge Digitizer* allow shifting axis marks and updating the coordinates retrieved. When using *digitize*, *g3data*, *WebPlotDigitizer* or *ycasd*, axis points can be re-captured and the coordinates are automatically updated thereon. Apparently, *Plot Digitizer* removes all results when re-calibrating axes.

Figures of paper presentations are of particular concern since they must be scanned or photocopied prior to data capturing which might result in distorted or skewed diagrams. Hence, we are interested how the tools perform if the figure would be rotated (see Additional file 4), and additionally being skewed (see Additional file 5), leading to non-orthogonal axes. *Digitize* does not correct for any distortion, *Plot Digitizer* accounts for rotation only. All other tools can deal with non-orthogonal axes.

#### Output format

Captured data must be exported to a format required for further analyses. All tools display delimiter based outputs. *Digitize* stores the output into an *R* data frame. Then, export to other kinds of data files is possible using appropriate R-functions. Similarly, the *R* matrix output of *ycasd* can be converted to a certain file format utilising *R. Ycasd* can also export a *Matlab* matrix (also delimiter based), which we prefer for our modelling. Beside delimiter based output, *Plot Digitizer* provides an XML file comprising extracted data points and calibration details. Results from *WebPlotDigitizer* can be forwarded to *plotly*, an online analysis and visualisation tool [18].

With all tools, we were successful in capturing the data points from the undistorted JPG (Additional file 3). To get a feeling for the default output, we stored the extracted data points in an Excel sheet (Additional file 6). During import of the results into Excel, the column delimiter was removed and the decimal delimiter was transformed to a dot.

### *Ycasd* in action

*Ycasd* was extensively used in our own scientific work regarding models of human haematopoiesis under chemotherapy and growth-factor applications. For our modelling, detailed time series data of blood parameters such as blood cell counts or cytokine concentrations under different clinical scenarios are required to estimate unknown model parameters. This is done by optimising the agreement of model predictions and data or to validate model predictions. *Ycasd* allowed us to establish a sufficiently rich data base for our modelling purposes. About N = 36 publications comprising about N = 186 graphical representations were processed with *ycasd* resulting in more than 3,500 collected data points. An overview of retrieved data is presented in Additional file 7.

## Discussion

Constructing dynamical mathematical models of biological or medical processes requires time series of clinical data in order to calibrate the models, estimate unknown parameters or validate model predictions. Since access to raw data is hardly possible in most of the cases, these data must be retrieved from the literature. Motivated by our own modelling work on human haematopoiesis for which we exploited data from numerous clinical settings, we developed *ycasd*, a tool for retrieving data points from figures of electronically available or scanned publications for which no raw data are available. In comparison to visual estimation or utilisation of a ruler, this kind of electronic data retrieval is more convenient, accurate and faster. In this paper, we provided an overview of the programme functions, and demonstrated how to use the tool on an example.

A couple of other tools are available for capturing data points from figures [8]. We compared the functionality of *ycasd* with other free software with emphasis on practical issues. Observed differences probably mirror different key applications which the developers had in mind when developing the tools. However, these intentions were scarcely communicated. In general, tools which were developed for certain needs do not provide the same functional range as others, see also the discussion in [19].

*Ycasd* was developed to fulfil our needs regarding capturing data from a variety of graphical representations; most importantly time series data with many data points required for modelling purposes. Therefore, the following key issues are important for our application: applicability to many kinds of graphical representations, easy accessibility of major functions, convenient calibration of diagrams, quick data collection with options for corrections and error estimation and export to required data formats. *Ycasd* was optimised regarding these issues.

The most apparent aspect is that our tool, in contrast to all others, does not require a certain input file format to open and process figures. When using *ycasd*, figures only have to be displayed with one’s preferred viewer and pixels will be captured from the screen independently of the viewer or the file format of the figure. An alternative way of handling arbitrary graphic formats is to import screenshots via clipboard which is featured by some tools (*Engauge Digitizer*, *WebPlotDigitizer*).

All functions of *ycasd* can be reached via a single window. This simplifies the usage of the software and speeds up the process of data collection as much as possible. On the other hand, this restricts its functionality.

We spend some efforts to deal with rotated and skewed figures which we often encountered in paper presentations. While rotation is a typical result of copying papers, skewness of presentations is of minor concern. Most of the tools considered can deal with both, rotation and skewness.

Since user mistakes occur frequently during data retrieval, we also optimised the way how to correct these. This applies for both, corrections of axes and data points. *Ycasd* supports deletion, insertion and overwriting of data points and axes calibrations at any time during analysis without losing collected data. A comparable convenience regarding this issue is offered by *Engauge Digitizer*.

If one aims at performing statistical analysis of data, capturing error might be of concern. Most tools display some kind of capturing errors, but it is unclear how they were calculated and therefore we neglected this issue for our comparisons.

We have to acknowledge that most tools allow more or less convenient workarounds of the above mentioned functions. For example, if not available, raw pixel coordinates can be retrieved by an appropriate definition of axes. Correction of points can often be performed by deleting the data point, appending the corrected version and re-ordering. For some tools, only specific delimiters can be chosen for output. But it would be easy to generate an arbitrary delimiter by copying the output to a text editor and replacing it. In consequence, none of the tools lack essential functionality required for data retrieval. Conversely, none of the tools can deal with every situation which might occur in figure presentations. Especially problematic are irregularities of axes for example interruptions of scales or non-equidistant scales. This might require manual corrections of retrieved data. Since we are both, developers and users of the software, we believe that our solution is a good compromise between functionality and requirements for medium-scale data retrieval from the literature. We used *ycasd* successfully for extracting a large number of data points from figures of several publications (see Additional file 7) to establish a data base for our modelling purposes [1-3]. We therefore believe that our tool could also be of significant value for other groups with similar research interests. We also believe that *ycasd* can be useful for other applications requiring data retrieval from literature such as systematic reviews and meta-analyses or to reconstruct survival time data [20].

Automated data retrieval would be a qualitative improvement. Indeed, there are some efforts regarding this issue. *Dexter*, *Engauge Digitizer* and *WebPlotDigitizer* support automatic detection of axes, of curves or of points. *Plot Digitizer* invokes the external tool *AutoTrace*[21] for automatic curve detection. However, this approach is technically challenging as also acknowledged by the developers. We experienced mixed quality of automated data retrievals. Further improvements are desirable, especially with respect to reliability in different situations (e.g. complex graphs, figures with multiple features to be collected).

## Conclusions

We conclude that we developed an easy to use software tool which is suitable for quick and convenient data retrievals from graphical representations such as papers. The software could be useful for many kinds of analyses for which published data are required but are not available in raw formats.

## Availability and requirements

**Project name**: *ycasd*

**Project home page**: https://sourceforge.net/projects/ycasd/

**Operating systems(s)**: Microsoft Windows

**Programming language**: C, Assembly

**Other requirements**: none

**License**: GNU General Public License version 3 [16]

**Any restrictions to use by non-academics**: GNU General Public License version 3

### Development

Our tool and its source code are freely available. It can be modified to suit certain needs. The binaries of the latest version 1.5 of *ycasd* were compiled with *MinGW* (GNU Compiler collection version 4.7.2, GNU Binutils version 2.23.1) [22]. The binaries are located in Additional file 2 and the source files reside in Additional file 8.

## Competing interests

The authors declare that they have no competing interests.

## Authors’ contributions

AG and MS wrote the paper. SS contributed to writing and discussion. AG, MS and SS developed the programme. AG implemented the programme. All authors read and approved the final manuscript.

## Supplementary Material

Additional file 1 **Scanned example.** The file contains an example of a distorted figure due to scanning. To achieve this, we scanned our paper draft several times after printing and selected one of the poor copies. Click here for file

Additional file 2 ***Ycasd* package.** The zip archive contains the binary, dynamic link library, pdf with sample figure, quick guide, readme file and license information. Click here for file

Additional file 3 **Sample JPG.** JPG version of the sample figure used for testing the tools. Click here for file

Additional file 4 **Sample JPG rotated.** JPG version of the sample figure which is rotated by 18° counter-clockwise. Click here for file

Additional file 5 **Sample JPG rotated and skewed.** JPG version of the sample figure which is rotated by 18° counter-clockwise and horizontally skewed. Click here for file

Additional file 6 **Table of extracted data points.** Table of data points of our sample JPG extracted with the specified capturing tools. Click here for file

Additional file 7 ***Ycasd* in action.** The document provides an overview of the number of figures and data points which we collected by our tool. Click here for file

Additional file 8 ***Ycasd* source code.** The zip archive provides all sources which are needed to build the binaries of *ycasd*. Click here for file
