# Supplementary material for: Ycasd – a tool for capturing and scaling data from graphical representations
Source: BMC Bioinformatics. 2014 Jun 25;15:219. doi: 10.1186/1471-2105-15-219 (PMC4085079; doi:10.1186/1471-2105-15-219)
Supplement: Additional file 1 — Scanned example. The file contains an example of a distorted figure due to scanning. To achieve this, we scanned our paper draft several times after printing and selected one of the poor copies. [file 1471-2105-15-219-S1.pdf]

2. Schirm S, Engel C, Loeffler M, Scholz M: **A Biomathematical Model of Human Erythropoiesis under Erythropoietin and Chemotherapy Administration.** *PLoS ONE* 2013, **8**:e65630.

3. Scholz M, Schirm S, Wetzler M, Engel C, Loeffler M: **Pharmacokinetic and - dynamic modelling of G-CSF derivatives in humans.** *Theoretical Biology and Medical Modelling* 2012, **9**:32.

4. **MATLAB - The Language of Technical Computing** [http://www.mathworks.com/products/matlab/].

5. Ihaka R, Gentleman R: **R: A Language for Data Analysis and Graphics.** *Journal of Computational and Graphical Statistics* 1996, 5:299.

6. **Ghostscript and GSview** [http://pages.cs.wisc.edu/~ghost/].

7. **Converting scanned graphs to data** [http://en.wikipedia.org/wiki/Converting_scanned_graphs_to_data].

8. Gross A, Ziepert M, Scholz M: **KMWin – A Convenient Tool for Graphical Presentation of Results from Kaplan-Meier Survival Time Analysis.** *PLoS ONE* 2012, **7**:e38960.

9. **WineHQ - Run Windows applications on Linux, BSD, Solaris and Mac OS X** [http://www.winehq.org].

10. **Licenses - GNU Project - Free Software Foundation (FSF)** [http://www.gnu.org/licenses/].

11. **Minimalist GNU for Windows** [http://www.mingw.org/].

Figures

Figure 1 - ***Ycasd*** **interface window**

Representation of *ycasd* interface window. Settings apart from default as well as outputs are shown in black. White letters on grey background are labels for: a-"Define axes" area, b-Pixels p1 to p4 corresponding to axis intercepts, c-Values corresponding to axis intercepts, d-"Reset p*" button, e-Logarithmic y-Axis selector, f-Estimated clicking error, g-"Start/Stop capturing" button, h-"New data set" button, i-"Define output" area, j-Output matrix type selector, k-Output accuracy, m-Output text box.

Figure 2 – Sample figure

Figure 3 - Vector representation

Pixels p_1 to p_4 define the coordinate system and correspond to axis intercepts y_2, y_1, x_1, and x_2, respectively. For each new pixel p_5 coordinates x_3, y_3 will be calculated by *ycasd*.

Additional files

Additional file 1 – ***Ycasd*** **package**

The zip package contains the binary, dynamic link library, pdf with sample figure, quick guide, readme file and license information.

Additional file 2 – ***Ycasd*** **source code**

The zip archive provides all sources which are needed to build the binaries of *ycasd*.

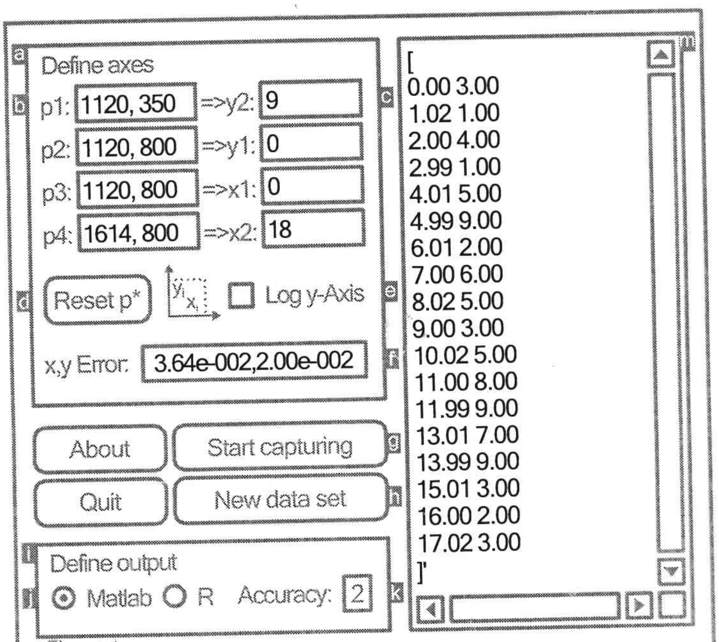

Figure 1

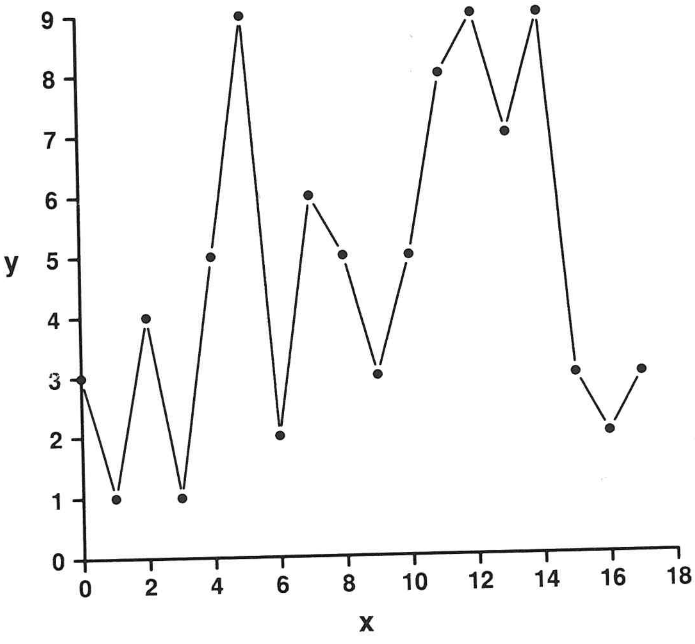

Figure 2
