# Supplementary figures and images for: Ycasd – a tool for capturing and scaling data from graphical representations
Source: BMC Bioinformatics. 2014 Jun 25;15:219. doi: 10.1186/1471-2105-15-219 (PMC4085079; doi:10.1186/1471-2105-15-219)

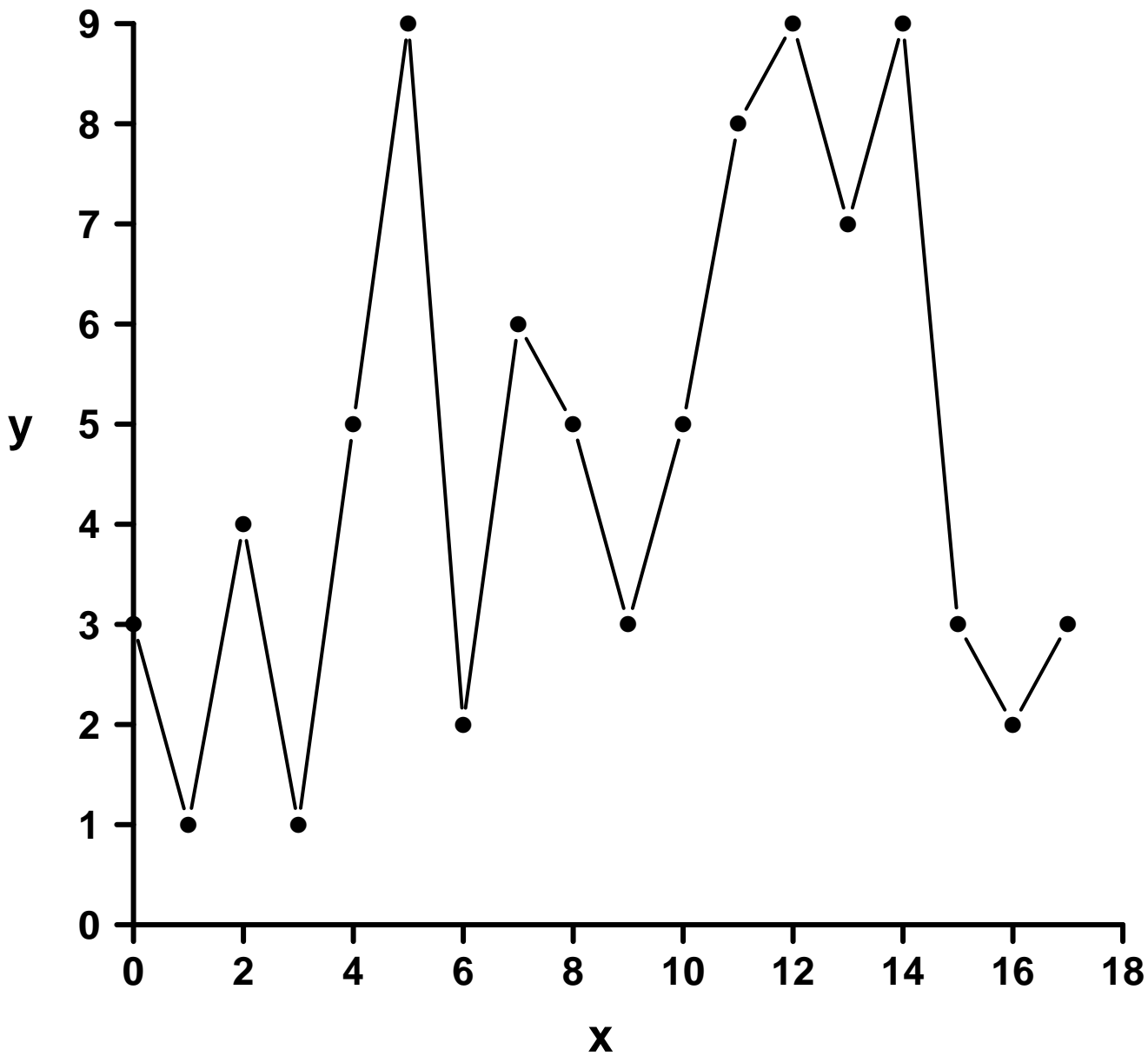

Supplement: Additional file 2 — Ycasd package. The zip archive contains the binary, dynamic link library, pdf with sample figure, quick guide, readme file and license information. [file 1471-2105-15-219-S2.zip › example.pdf]

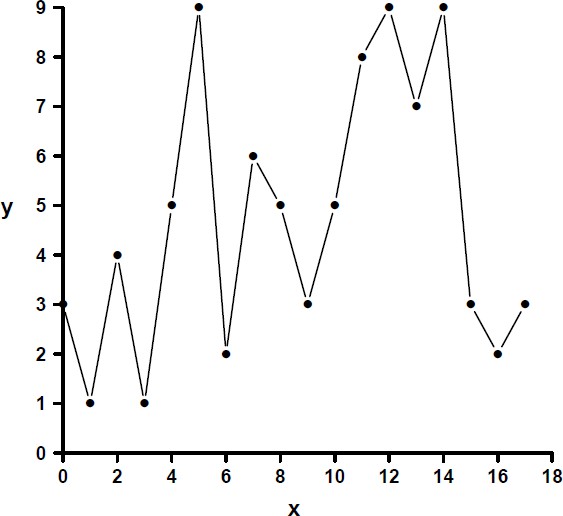

Supplement: Additional file 3 — Sample JPG. JPG version of the sample figure used for testing the tools. [file 1471-2105-15-219-S3.jpeg]

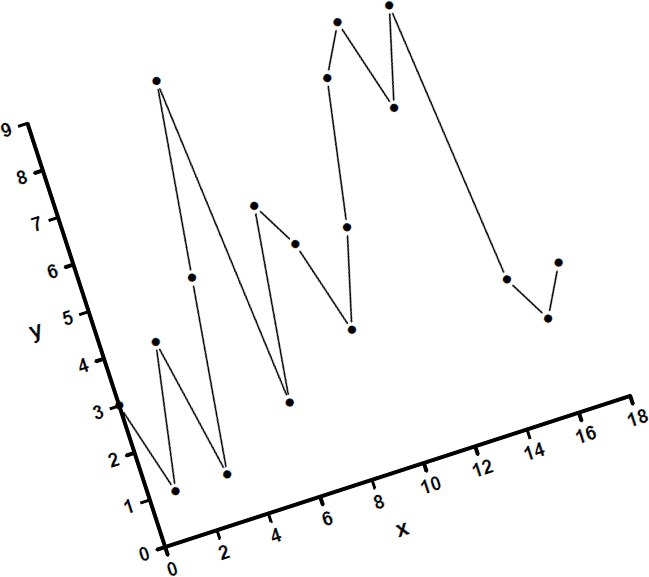

Supplement: Additional file 4 — Sample JPG rotated. JPG version of the sample figure which is rotated by 18° counter-clockwise. [file 1471-2105-15-219-S4.jpeg]

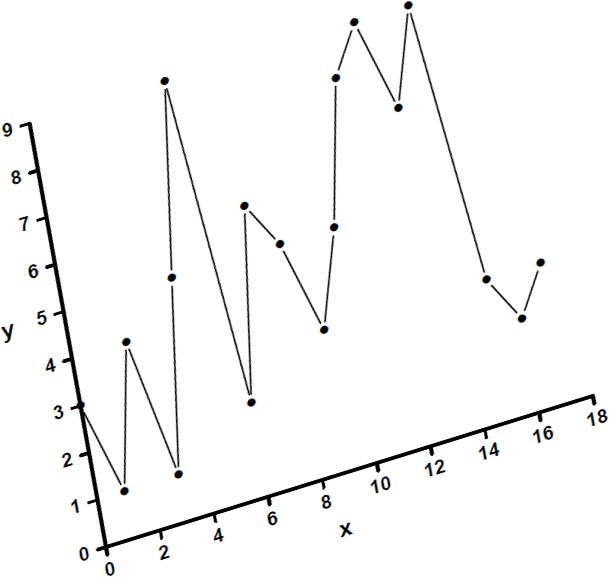

Supplement: Additional file 5 — Sample JPG rotated and skewed. JPG version of the sample figure which is rotated by 18° counter-clockwise and horizontally skewed. [file 1471-2105-15-219-S5.jpeg]
